# A Genome‐Wide Association Study of Colorectal Cancer Mortality Outcomes Among Individuals of African and Admixture Ancestry

**DOI:** 10.1002/mc.70086

**Published:** 2026-01-29

**Authors:** Thomas Lawler, Jirong Long, Rene Welch, Irene Ong, Oluwatoyosi Ogunmuyiwa, Rida A. Khatri, Martha Shrubsole, Shaneda Warren Andersen

**Affiliations:** ^1^ School of Medicine and Public Health, Carbone Cancer Center University of Wisconsin–Madison Madison Wisconsin USA; ^2^ Department of Medicine, Vanderbilt Epidemiology Center, Vanderbilt‐Ingram Cancer Center Division of Epidemiology Nashville Tennessee USA; ^3^ Department of Biostatistics and Medical Informatics, School of Medicine and Public Health University of Wisconsin–Madison Madison Wisconsin USA; ^4^ Department of Obstetrics and Gynecology, UW‐Health Hospital University of Wisconsin–Madison Madison Wisconsin USA; ^5^ Department of Population Health Sciences, School of Medicine and Public Health University of Wisconsin–Madison Madison Wisconsin USA

**Keywords:** Black people, colorectal neoplasms, health status disparities, race factors

## Abstract

African Americans have the highest colorectal cancer (CRC) mortality rates in the United States. We performed the first genome‐wide association study (GWAS) of overall and CRC‐specific mortality among African Americans with incident CRC to identify genetic contributors to CRC outcomes. Participants enrolled in the Southern Community Cohort Study in 2002–2009; incident CRC and mortality were identified via state cancer registries and the National Death Index. SNPs were genotyped across the genome via Illumina platforms and imputed using the Michigan Imputation Server with Minimac4. Associations with mortality were estimated as hazard ratios (HRs) with 95% confidence intervals (CIs) using Cox proportional hazards models, adjusted for age, sex, stage, and five principal components for ancestry. In total, 500 Black‐identifying participants were analyzed, including 316 deaths and 184 CRC‐specific deaths. Two novel loci in linkage disequilibrium (*r* ^2^ = 1) within *LTBP1* were associated with higher CRC‐specific mortality risk: rs34071846 and rs12712337 (per allele HR: 2.74, CI: 1.91–3.92, *p* = 3.78 × 10^−8^). An additional variant mapped to a gene for a noncoding RNA was associated with CRC‐specific mortality: rs10103953 (per allele HR: 0.52, CI: 0.42–0.66, *p* = 2.03 × 10^−8^). One loci mapping to *MCTP2* was associated with lower overall mortality risk: rs7171579 (per allele HR: 0.59, CI: 0.50–0.71, *p* = 2.13 × 10^−8^). In conclusion, evidence from the present study supports *LTBP1* and *MCTP2* as important to CRC mortality.

**Abbreviations**
CAAPA: Consortium on Asthma among African‐ancestry Populations in the Americas
CRC: colorectal cancer
eQTL: expression quantitative trail loci
GWAS: genome‐wide association study
HWE: Hardy–Weinberg Equilibrium
MAF: minor allele frequency
NDI: National Death Index
SCCS: Southern Community Cohort Study
SNP: single‐nucleotide polymorphism
TCGA: The Cancer Genome Atlas
TNM: tumor, node, metastasis

## 1 Introduction

Colorectal cancer (CRC) is the second leading cause of cancer mortality in the United States, and it is estimated that CRC will cause nearly 53,000 deaths in 2025 [1]. Despite considerable progress in reducing the population burden of CRC through improvements in early detection and treatment, significant racial disparities have persisted [2]. Non‐Hispanic Black Americans have a 34% higher CRC mortality rate compared to the non‐Hispanic White population [3], attributable in part to lower socioeconomic status and more aggressive tumor characteristics [2]. Given the disproportionate mortality of CRC for Black Americans, it is necessary to ensure that emerging treatments and prognosis tools can benefit the most burdened populations.

Emerging research has demonstrated that germline genetic variants may affect tumor characteristics that impact cancer progression and survival [4, 5, 6, 7, 8]. Consequently, there is interest in utilizing germline genetic markers in a clinical setting to inform prognosis or treatment selection [9]. Recent genome‐wide association studies (GWAS) have identified single‐nucleotide polymorphisms (SNPs) that may impact mortality or survival outcomes after a CRC diagnosis for specific clinical populations [10, 11, 12, 13, 14, 15, 16, 17, 18, 19, 20, 21]. In total, ~200 loci associated with CRC mortality or recurrence outcomes have been reported. Although the results have been inconsistent between studies, these findings may eventually support the development of precision medicine approaches for CRC. Importantly, previous studies were performed in predominantly European‐ or Asian‐ancestry samples [10, 11, 12, 13, 15, 16, 17, 18, 19, 20, 21]. African‐ancestry individuals have a unique genetic architecture with smaller haplotype blocks and differences in minor allele frequency (MAF) [22] than other ancestry populations meaning that previously published GWAS results may not generalize to the African‐ancestry population. Notably, Penney and colleagues used data from a population including individuals of European ancestry and African ancestry and identified a single variant (rs76766811) that showed an African‐ancestry‐specific association with increased risk for overall mortality (per allele hazard ratio [HR] 2.82, 95% confidence interval [CI]: 1.88–4.23) among African American participants with Stage II–III colon cancer receiving treatment in a clinical trial [14]. The variant is common among African‐ancestry populations but is rare in the European‐ and Asian‐ancestry populations, highlighting the need for diversity in biomedical research [14].

Performing studies with samples enriched for participants with African ancestry ensures that genetic discoveries support precision medicine approaches that benefit all individuals. Although previous studies have identified CRC risk variants in African Americans [23], we aim to complete the first GWAS of CRC mortality outcomes in a cohort of individuals who self‐identify as Black or African–American. We hypothesize that common germline genetic variants influence CRC mortality outcomes. Investigating genetic contributions in an African‐ancestry sample may identify novel variants not previously identified in predominantly European‐ and Asian‐ancestry samples. To accomplish this aim, we utilize data from the Southern Community Cohort Study (SCCS), a cohort with rich multilevel data designed to help address racial disparities in cancer outcomes.

## 2 Materials and Methods

### 2.1 SCCS Population

Data arise from 500 participants of the SCCS who developed incident CRC after enrollment. Briefly, the SCCS enrolled ~85,000 English‐speaking individuals aged 40–79 years during years 2002–2009 in 12 states in the southeastern United States. The majority of the cohort (86%) were enrolled from community health centers, while the remaining 14% were enrolled via telephone calls or general population mailings. Upon enrollment, participants completed questionnaires including demographic background, medical history and current medication use, family history of disease, smoking history, typical diet, and alcohol consumption. Additional information concerning SCCS recruitment, enrollment, and study design have been published previously [24]. In total, 46% of participants provided a blood sample at enrollment, which was stored at −80°C. All study activities conformed to the Declaration of Helsinki and all participants provided written informed consent. The Institutional Review Boards of Vanderbilt University Medical Center and Meharry Medical College provided approval for this study.

### 2.2 Identification of Incident Colorectal Cancer and Mortality Outcomes

Participants who developed incident CRC after enrollment were identified via linkages with state cancer registries and the National Death Index (NDI). Incident CRC was defined using International Classification of Diseases for Oncology, 3rd Edition (ICD‐O‐3) codes C180–C189, C199, and C209. Linkage to state cancer registries was complete through 2016–2019, registry dependent. Each participant's tumor, node, metastasis (TNM) summary stage (*American Joint Committee on Cancer, Seventh Edition*) [25] at diagnosis was also identified via state registries. Mortality outcomes (overall mortality and CRC‐specific mortality) were identified from the NDI. Linkage with the NDI was complete through December 31, 2020.

### 2.3 Assessment of Genetic Variation and Quality Control Procedures

Data on genetic variants arise from previously described projects [26, 27, 28, 29, 30]. Participants were genotyped using three different Illumina platforms with genome‐wide coverage, with a preimputation genotyping rate ranging from 96.6% to 99.9%. There were no duplicated samples across studies. Each data set was imputed using the Michigan Imputation Server with Minimac4 [31]. Prior to imputation, each variant's chromosome and base‐pair location was converted to Genome Reference Consortium Human Build 37 (GRCh37), as needed, using the Liftover tool from the University of California Santa Clara Genome Browser (https://genome.ucsc.edu) [32]. Variant identifiers were made consistent across studies using the chromosome:location format, and all nonautosomal variants were excluded. Consistent with guidelines from the Michigan Imputation Server [31], the following classes of variants were excluded from each data set prior to imputation: insertion/deletion polymorphisms (indels); SNPs with high‐missingness (≥ 10%), mono‐allelic SNPs; SNPs with more than two alleles; and ambiguous SNPs. For duplicate SNPs, only the first instance was retained in the data set. Allele mismatches and strand flips were identified via comparison to the reference panel and corrected prior to imputation. No samples were identified with a SNP‐missingness rate of ≥ 5%. Data were imputed using the Consortium on Asthma among African‐ancestry Populations in the Americas (CAAPA) reference panel. Only SNPs with imputation quality score of *R* ^2^ ≥ 0.30 were retained in the data set.

After imputation, the eight data sets were merged to create an analysis data set. After merging, SNPs were excluded that met at least one of the following criteria: missingness ≥ 5% (82,090,042 SNPs), Hardy–Weinberg Equilibrium (HWE) *p* value < 5.0 × 10^−8^ (80 SNPs), or MAF < 0.05 (2,155,075 SNPs). Variants with MAF < 0.05 were excluded because these rare variants may have lower imputation accuracy [33] and because statistical power to identify associations for rare variants is limited [34]. In total, 6,463,067 SNPs remained and were included in the analysis. All quality control procedures were performed using PLINK versions 1.9 and 2 [35] and bcftools version 1.20 [36]. Pairwise identity‐by‐descent was utilized to assess relatedness between individuals; no individuals were related in this sample. Further, ancestry analysis was performed using ADMIXTURE software [37] with the 1000 genomes reference panel.

### 2.4 Inclusion and Exclusion Criteria

The inclusion criteria for the present analysis were incident CRC and self‐reported Black racial identity (*N* = 832). Participants were excluded who did not provide a blood sample at enrollment or did not consent to genetic analysis (*N* = 305), or who were diagnosed with CRC in situ (*N* = 22). Five participants were excluded who were determined to have been diagnosed with anal cancer. In total, 500 participants with data on genome‐wide genetic variation were included in the analysis.

### 2.5 The Cancer Genome Atlas Data Set

As a replication data set, clinical and genomic data for 65 Black‐ or African American‐identifying participants in The Cancer Genome Atlas (TCGA) colorectal adenocarcinoma and rectal adenocarcinoma data sets were downloaded from the Genomic Data Commons Data Portal [38]. Ancestry analysis was completed using ADMIXTURE [37] with the 1000 genomes reference panel.

### 2.6 Statistical Analysis

Associations between genome‐wide genetic variants and overall, CRC‐specific mortality were estimated via Cox proportional hazards models using an additive genetic model and expressed as HRs with 95% CIs per one minor allele increase. Follow‐up time was defined as the duration of time (in months) from a participant's age at CRC diagnosis until their age at mortality or the end of follow‐up in the NDI (i.e., 12/31/2020). For participants whose age at CRC diagnosis and mortality were equivalent, the survival time was increased to 1 month. All models were adjusted for age at CRC diagnosis, sex (male, female), TNM stage at diagnosis (I, II, III, IV, missing), and the first five principal components. A threshold of *p* < 5.0 × 10^−8^ was utilized to indicate statistical significance. Because GWAS analysis may be underpowered to detect SNPs with modest associations [39], SNPs meeting the threshold of *p* < 5.0 × 10^−6^ were also reported, as these variants may be of interest for replication in independent cohorts. GWAS analysis was performed using the plinkCoxSurv function from the gwasurvivr package in R [40]. Principal components were calculated using PLINK version 1.9. Manhattan plots were created using the ggplot2 package in R. QQ plots were created using the qqman package in R [41].

For SNPs reaching genome‐wide statistical significance in the SCCS data set, a stratified analysis was performed by tumor location (colon vs. rectum). Further, we attempted to replicate the associations in an independent sample of Black‐ or African‐American identifying participants with colon or rectal adenocarcinoma from the TCGA (*N* = 65). In the replication data set, associations were limited to overall mortality only. Due to limitations in sample size, associations were adjusted for age at CRC diagnosis, sex, and stage only.

For SNPs reaching genome‐wide statistical significance in the SCCS data set, gene annotations were obtained from the online dbSNP database [42]. Corresponding gene expression in the colon was obtained from the Genotype‐Tissue Expression (GTEx) online database [43]. GTEx was also used to determine whether identified SNPs are expression quantitative trait loci (eQTLs). The Sorting Intolerant From Tolerant (SIFT) [44] and PolyPhen2 [45] online databases were searched to predict the impact of identified variants on the amino acid sequence and function of corresponding proteins.

SNPs previously linked to CRC mortality or survival outcomes in GWAS were identified from the National Human Genome Research Institute and European Bioinformatics Institute (NHGRI‐EBI) GWAS catalog [46]. Summary statistics were downloaded from the NHGRI‐EBI GWAS Catalog on 12/18/2024 for references [10, 11, 12, 13, 14, 15, 16, 17, 18, 19, 20]. Statistically significant associations were also extracted from the recent publication by Wills et al. [21]. For these variants, associations with overall and CRC‐specific mortality in the present sample are reported. For variants that were not included in our analysis, a suitable proxy variant in linkage disequilibrium for the African‐ancestry population was identified via the LDlinkR package [47], when available. In total, there were 123 independent associations reported for overall or CRC‐specific survival. A total of 79 associations were reported for time to CRC recurrence of progression‐free survival. For these previously reported SNPs (*N* = 202), associations were available for 141 (or for a suitable proxy) in the present sample. There were 61 SNPs for which results were not available which are mono‐allelic in the African‐ancestry population or were not included due to low MAF (< 0.05) or low imputation quality in the present sample.

## 3 Results

### 3.1 Participant Characteristics

A majority of participants were female (60%) and had annual household incomes < $15,000 (61%) (Table 1). The median (interquartile range) age at diagnosis was 60 (54–68) years. The median time between CRC diagnosis and mortality or censoring was 51 (12–110) months. In total, 316 participants (63%) experienced overall mortality and 184 participants (37%) experienced CRC‐specific mortality before the end of follow‐up. The ancestry in this sample was 87% African (range: 3%–100%), 12% European (range: 0%–91%), and < 1% each East Asian, South Asian, and Admixed American ancestry.

**Table 1:** Participant characteristics (*N* = 500)^a^.

		Overall mortality	
Characteristics	Full sample (*N* = 500)	Alive (*N* = 184)	Deceased (*N* = 316)
Age at CRC dx. (years), median (IQR)	60 (54–68)	59 (53–65)	61 (55–69)
Age at CRC dx. (years), categorized			
40–49	46 (9%)	20 (11%)	26 (8%)
50–54	81 (16%)	38 (21%)	43 (14%)
55–59	105 (21%)	37 (20%)	68 (22%)
60–64	89 (18%)	40 (22%)	49 (16%)
65–69	77 (15%)	23 (13%)	54 (17%)
≥ 70	102 (20%)	26 (14%)	76 (24%)
Enrollment source			
Community health centers	468 (94%)	166 (90%)	302 (96%)
Mail/telephone	32 (6%)	18 (10%)	14 (4%)
Sex			
Female	299 (60%)	115 (63%)	184 (58%)
Male	201 (40%)	69 (38%)	132 (42%)
Education			
Missing	9 (2%)	5 (3%)	4 (1%)
< HS graduate	184 (37%)	53 (29%)	131 (41%)
HS graduate	169 (34%)	64 (35%)	105 (33%)
Post‐HS education	138 (28%)	62 (34%)	76 (24%)
Annual household income			
Missing	14 (3%)	7 (4%)	7 (2%)
< $15,000/year	303 (61%)	94 (51%)	209 (66%)
$15,000–49,999/year	160 (32%)	67 (36%)	93 (29%)
≥ $50,000/year	23 (5%)	16 (9%)	7 (2%)
Tumor diagnosis stage			
I	105 (21%)	65 (35%)	40 (13%)
II	94 (19%)	41 (22%)	53 (17%)
III	83 (17%)	34 (18%)	49 (16%)
IV	99 (20%)	6 (3%)	93 (29%)
Missing	119 (24%)	38 (21%)	81 (26%)
Tumor location			
Missing	29 (6%)	0 (0%)	29 (9%)
Colon	362 (72%)	142 (77%)	220 (70%)
Rectum	109 (22%)	42 (23%)	67 (21%)

Abbreviations: CRC, colorectal cancer; HS, high school; IQR, interquartile range.

^a^ Data presented as median (interquartile range) for continuous variables, and as *N* (%) for categorical variables.

### 3.2 Associations Between Single‐Nucleotide Polymorphisms and Mortality

**Figure 1:**
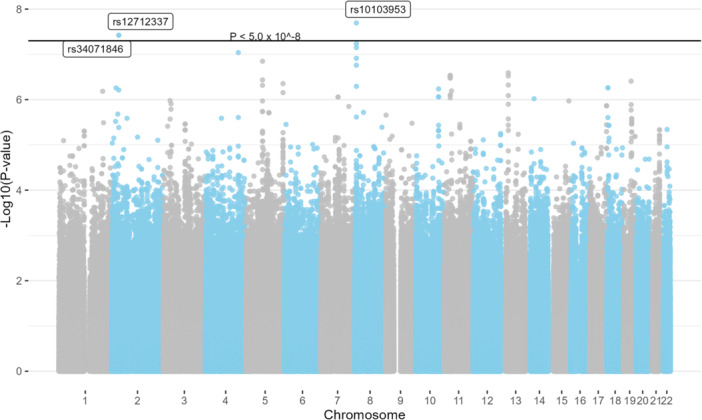
Manhattan plots for associations between single‐nucleotide polymorphisms and colorectal cancer‐specific mortality among individuals with incident colorectal cancer in the Southern Community Cohort Study (*N* = 500).

**Figure 2:**
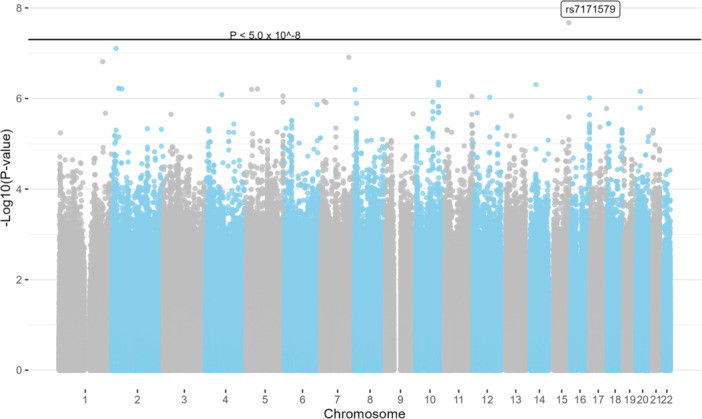
Manhattan plots for associations between single‐nucleotide polymorphisms and overall mortality among individuals with incident colorectal cancer in the Southern Community Cohort Study (*N* = 500).

Three SNPs were associated with risk for CRC‐specific mortality: rs34071846 (*LTBP1*, per allele HR 2.74, CI: 1.91–3.92); rs12712337 (*LTBP1*, per allele HR 2.74, CI: 1.91–3.92); and rs10103953 (*LOC124901866*, per allele HR 0.52, CI: 0.42–0.66, Table 2 and Figure 1). There were 144 SNPs that were associated with CRC‐specific mortality at the threshold *p* value < 5.0 × 10^−6^ (Supporting Information S2: Table 1). One SNP was associated with overall mortality: rs7171579 (*MCTP2*, per allele HR 0.59, CI: 0.50–0.71, Table 2 and Figure 2). There were 88 SNPs that were associated with risk for overall mortality at the threshold *p* value < 5.0 × 10^−6^ (Supporting Information S2: Table 2). For both mortality outcomes, the distribution of *p* values showed modest deviance from the null distribution (Supporting Information S1: Figure 1), which may be related to linkage disequilibrium. In stratified analysis, the association between *LTBP1* variants and CRC‐specific mortality was stronger for rectal cancer (per allele HR 3.78, CI: 1.68–8.53) compared to colon cancer (per allele HR 2.21, CI: 1.32–3.70) (*p*‐interaction = 0.05, Table 3). A similar interaction was observed for the association between *MCTP2* variant rs7171579 and overall mortality (*p*‐interaction = 0.001, Table 3).

**Table 2:** Summary of associations between single‐nucleotide polymorphisms and mortality outcomes reaching genome‐wide statistical significance (*p* value < 5.0 × 10^−8^) among individuals with incident colorectal cancer (*N* = 500).

	Chr:position^a^	Gene^a^	Alleles (Major/Minor)	Sample MAF	HR^b^ (95% CI)	*p*
CRC‐specific mortality^a^						
rs34071846	2:33314964	*LTBP1*	C/A	0.07	2.74 (1.91–3.92)	3.78 × 10^−8^
rs12712337	2:33315230	*LTBP1*	C/T	0.07	2.74 (1.91–3.92)	3.78 × 10^−8^
rs10103953	8:8834240	*LOC124901866*	G/T	0.45	0.52 (0.42–0.66)	2.03 × 10^−8^
Overall mortality^a^						
rs7171579	15:94838899	*MCTP2*	C/T	0.30	0.59 (0.50–0.71)	2.13 × 10^−8^

Abbreviations: CI, confidence interval; CRC, colorectal cancer; HR, hazard ratio; MAF, minor allele frequency.

^a^ rsIDs and variant chromosome, position, and gene are from the Genome Reference Consortium human reference genome GRCh37.

^b^ All hazard ratios are per one minor allele increase, adjusted for age at diagnosis, sex, stage, and the first five principal components.

**Table 3:** Summary of associations between single‐nucleotide polymorphisms and mortality outcomes reaching genome‐wide statistical significance (*p* value < 5.0 × 10^−8^) among individuals with incident colorectal cancer, stratified by tumor location (*N* = 471).

Colon cancer (*N* = 362)	Chr:position^a^	Gene^a^	Alleles (Major/Minor)	Sample MAF	HR^b^ (95% CI)	*p*
CRC‐specific mortality^a^						
rs34071846	2:33314964	*LTBP1*	C/A	0.06	2.21 (1.32–3.70)	0.003
rs12712337	2:33315230	*LTBP1*	C/T	0.06	2.21 (1.32–3.70)	0.003
rs10103953	8:8834240	*LOC124901866*	G/T	0.45	0.55 (0.41–0.74)	5.45 × 10^−5^
Overall mortality^a^						
rs7171579	15:94838899	*MCTP2*	C/T	0.29	0.72 (0.58–0.90)	0.003
Rectal cancer (*N* = 109)						
CRC‐specific mortality^a^						
rs34071846	2:33314964	*LTBP1*	C/A	0.06	3.78 (1.68–8.53)	0.001
rs12712337	2:33315230	*LTBP1*	C/T	0.06	3.78 (1.68–8.53)	0.001
rs10103953	8:8834240	*LOC124901866*	G/T	0.51	0.44 (0.25–0.76)	0.004
Overall mortality^a^						
rs7171579	15:94838899	*MCTP2*	C/T	0.33	0.36 (0.23–0.56)	4.48 × 10^−6^

Abbreviations: CI, confidence interval; CRC, colorectal cancer; HR, hazard ratio; MAF, minor allele frequency.

^a^ rsIDs and variant chromosome, position, and gene are from the Genome Reference Consortium human reference genome GRCh37.

^b^ All hazard ratios are per one minor allele increase, adjusted for age at diagnosis, sex, stage, and the first five principal components.

**Figure 3:**
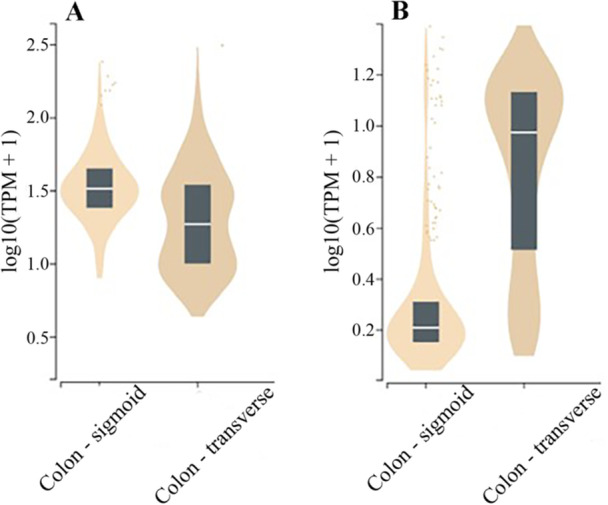
(A, B) mRNA expression of *LTBP1* and *MCTP2* in the sigmoid and transverse colon.^a^ ^a^Gene expression data obtained from the Genotype‐Tissue Expression (GTEx) portal. Data presented as median value with inter‐quartile range. TPM, transcripts per million.

For the identified SNPs (*N* = 4), no impact on the amino acid sequence or protein function were identified via SIFT or PolyPhen2. However, *MCTP2* and *LTBP1* are both expressed in the sigmoid and transverse colon (Figure 3). Both rs34071846 and rs12712337 were identified as eQTLs for *LTBP1* in the adrenal gland, while rs7171579 was identified as an eQTL for *MCTP2* in the transverse colon.

### 3.3 Replication Analysis in The Cancer Genome Atlas

Among TCGA participants, the median [IQR] at age diagnosis was 61 [51–69] years. The ancestry in this sample was 83% African (range: 32%–100%), 13% European (range: 0%–39%), 3% Admixed American (range 0%–32%), and < 1% each for East Asian and South Asian ancestry. Sample size limited analyses of the TCGA data in that 14 of the 65 participants died during the study period. Specifically, associations between the two variants in *LTBP1* (rs34071846 and rs12712337) were not estimable due to small sample size and low MAF (MAF = 0.05). Variant rs7171579 in *MCTP2* observed a similar association as compared to the SCCS data set (per allele HR 0.65, CI: 0.25–1.66, *p* = 0.37, Table S3). For rs10103953 (*LOC124901866*), an association with higher risk for overall mortality was observed (per allele HR 4.45, CI: 1.22–16.26, *p* = 0.02). For this association, 2 of 10 participants with the homozygous recessive genotype (T/T) were deceased, compared to 0 of 17 participants with the homozygous dominant genotype (G/G) and 12 of 38 participants who are heterozygous (G/T).

### 3.4 Evaluation of Previously Reported Single‐Nucleotide Polymorphisms Within African‐Ancestry Populations

Penney and colleagues reported that variant rs76766811 was associated with lower overall survival (per allele HR 2.82, CI: 1.88–4.23, Supporting Information S4: Table 4) and disease‐free survival (per allele HR 2.27, CI: 1.62–3.18, Supporting Information S5: Table 5) among 358 African Americans with resected Stage II–III colon cancer receiving adjuvant therapy [14]. For this variant, no association with overall (per allele HR 0.96, CI: 0.77–1.18) or CRC‐specific mortality (per allele HR 0.89, CI: 0.66–1.18) was observed in the present sample. Likewise, for 144 participants with Stage II–III colon cancer, rs76766811 was not associated with either outcome (overall mortality: per allele HR 1.12, CI: 0.70–1.80; colon cancer‐specific mortality: per allele HR 1.43, CI: 0.72–2.82). No other studies were identified that reported genome‐wide associations in a predominantly Black‐identifying or African ancestry sample.

### 3.5 Evaluation of Previously Reported Single‐Nucleotide Polymorphisms Within Non‐African‐Ancestry Populations

The majority of previous studies include European‐ or Asian‐ancestry samples [10, 11, 12, 13, 15, 16, 17, 18, 19, 20, 21]. For the SNPs reported in these studies, none were statistically significant in the present sample (*p* < 5.0 × 10^−8^). Further, there was no overlap with SNPs that were nominally significant (*p* < 5.0 × 10^−6^) for CRC‐specific mortality (Supporting Information S2: Table 1, *N* = 144) or overall mortality (Supporting Information S2: Table 2, *N* = 88). For a chromosome 5 variant (rs244509, mapped genes *WDR36/RPS3AP21*) that was associated with lower risk for overall mortality in a GWAS of 1926 predominantly European‐ancestry individuals with advanced CRC (per allele HR: 0.81, CI: 0.73–0.90) [18], we observed an association with lower risk for CRC‐specific mortality (Supporting Information S4: Table 4, per allele HR: 0.49, CI: 0.30–0.81, *p* = 0.005). We also identified modest associations with CRC‐specific survival (*p* ≤ 0.006) for several SNPs in chromosome 13 that were associated with disease‐free survival in a GWAS of 505 European‐ancestry individuals [19] (Supporting Information S5: Table 5), including three variants that were associated with higher risk (rs7319093, rs61965998, and rs9557921, per allele HR: 1.32–1.34 for all variants), and one variant that was associated with lower risk (rs7323315, per allele HR: 0.71, CI: 0.57–0.90). Associations with overall mortality were similar. Risk estimates were not available from the original publication for comparison.

## 4 Discussion

We report four germline genetic variants associated with overall or CRC‐specific mortality in a sample of Black‐identifying individuals diagnosed with incident CRC. This study adds to a growing body of evidence linking germline genetic variants to cancer outcomes. Importantly, GWAS results identify genes and biological pathways to target for intervention [48]. Further, GWAS may support the development of polygenic risk scores to improve cancer prognostication, and those most likely to respond to treatment [9]. This study builds on previous research by including a sample enriched for individuals with African ancestry, a population with unique genetic characteristics [22]. Including African‐ancestry individuals in GWAS ensures findings from genetic research generalize across populations, as well as identifies novel loci that impact disease outcomes [22].

We find two intronic variants in complete linkage disequilibrium mapping to *LTBP1* (rs34071846 and rs12712337) that are associated with a higher risk for CRC‐specific mortality. There is a stronger association with CRC‐specific mortality for participants with tumors in the rectum versus colon, which may reflect the distinct embryological origins of these tissues and differences in gene expression patterns [49], although this interaction is based on a limited number of rectal cancer cases. A recent study by Wills and colleagues reports an association between *LTBP1* variant rs62135742 and increased overall mortality (HR 1.80, CI: 1.42–2.29) among 514 participants with proximal colon tumors from the COIN and COIN‐B clinical trials, although this association did not replicate in the UK‐Biobank sample (*N* = 1433) [21]. Notably, this variant is in modest linkage disequilibrium with the *LTBP1* variants we identify (D′: 0.58 in the African‐ancestry population). While the variants we identify are not expected to impact protein function, they are both eQTLs for the adrenal glands, with the minor allele related to higher transcript expression. *LTBP1* is expressed in the colon [50] (Figure 3) and encodes latent transforming growth factor beta (TGF‐β) binding protein 1, an extracellular protein that binds to and supports the activation of TGF‐β [51]. TGF‐β is a well‐known driver of aggressive tumor characteristics in CRC, with higher activity linked to epithelial–mesenchymal transition, angiogenesis, metastasis, immunosuppression, and resistance to chemotherapy [52].

Previous research links higher tumor *LTBP1* mRNA expression to poor cancer outcomes [53, 54, 55, 56, 57, 58]. In CRC, a risk score based on the gene expression of six genes including *LTBP*1 is associated with lower overall survival, disease‐free survival, and progression‐free survival among patients with Stage II–III disease from the Gene Expression Omnibus repository [56]. Higher tumor *LTBP1* expression has also been linked to poor prognosis and aggressive tumor characteristics in other cancer subtypes [53, 54, 55, 57, 58]. Mechanistically, in vitro experimental studies demonstrate that *LTBP1* inhibition or knockout inhibits cell proliferation and invasion potential, decreases the mesenchymal phenotype, and increases sensitivity to 5‐fluorouracil treatment in gastric cancer and esophageal cancer cell lines [53, 54].

We also report an association between rs7171579, mapping to *MCTP2*, and lower risk for overall mortality. There is a similar association with CRC‐specific mortality. The association with overall mortality is similar in the TCGA replication sample, although not statistically significant. *MCTP2* is located in chromosome 15q26 and encodes multiple C2 domain transmembrane containing protein 2, linked to normal cardiac development and risk for congenital heart disease [59]. Currently, there is limited evidence linking *MCTP2* to the development or progression of cancer. However, Sun and colleagues report that higher expression of circular *MCTP2* RNA is related to higher sensitivity to cisplatin in gastric cancer cell lines and tissues, as well as better prognosis for gastric cancer patients [60]. Further, they confirm that circular *MCTP2* RNA is able to inhibit proliferation while promoting apoptosis in cisplatin‐resistant gastric cancer cells [60], providing a mechanistic understanding of how *MCTP2* expression influences gastric tumor development and treatment response. Further, Tanskanen and colleagues report the presence of a rare splice‐site variant in *MCTP2* among Finnish individuals with early‐onset CRC or familial CRC, suggesting that rare inactivating mutations in *MCTP2* may contribute to CRC incidence [61]. The rs7171579 variant is in the first intron of *MCTP2* and consequently is not predicted to impact the amino acid sequence or protein function. However, rs7171579 is an eQTL linked to higher *MCTP2* expression in the transverse colon, where expression levels are high. Currently, the biological impact of increased *MCTP2* expression in the colon or rectum has not been investigated.

Additionally, there is an association with lower CRC‐specific mortality risk for variant rs10103953 that maps to a gene for a noncoding RNA. To the authors' knowledge, the biological functions of this gene are unknown, and no associations for variants within this gene have been published. Noncoding RNAs are linked to CRC prognosis and response to treatment with 5‐fluorouracil, oxaliplatin, methotrexate, and cetuximab, making them potentially attractive targets for intervention [62]. Surprisingly, we observe that the rs10103953 variant is associated with a higher risk for overall mortality in the TCGA replication sample. Although this is unexpected, it may be related to the small replication sample size and limited number of deaths. Additional research is required to corroborate the observed association in an independent sample and to elucidate the biological role for the associated noncoding RNA.

Further, there are several dozen variants that are suggestively associated (criteria *p* value < 5.0 × 10^−6^) with overall or CRC‐specific mortality in our sample. These include variants in genes whose expression patterns or somatic mutations are linked to CRC prognosis and tumor characteristics in other samples, including *SORCS1* [63], *CACNA2D1* [64], *PVT1* [65], *DAB2IP* [66], and *RAB12* [67], as well as the potential tumor suppressor gene *OPCML* [68].

Emerging evidence from multiple cancers indicates that germline variants may influence prognosis [69], response to chemotherapy and immune checkpoint inhibitors [70, 71], characteristics of the tumor microenvironment (e.g., immune cell infiltration) [8], and the development of somatic mutations that drive tumor progression [4, 7]. Consequently, germline genetic research may support the development of precision medicine approaches to inform prognosis and/or the selection of cancer treatments [9]. However, challenges identifying genetic associations with cancer outcomes that generalize across populations limit the clinical impact of genetic findings. While there are 12 previously published GWAS that report genetic associations with CRC mortality or survival outcomes [10, 11, 12, 13, 14, 15, 16, 17, 18, 19, 20, 21], there are few associations that replicate across studies or in the present study of predominantly African‐ancestry participants. Specifically, we did not find any overlap between the previously identified SNPs and the genome‐wide statistically significant or nominally significant SNPs from the present study. In part, the overall lack of replication between studies may be explained by limitations in statistical power and false‐positive associations driven by small sample size for some studies. Sample sizes have varied considerably across studies from < 600 [10, 11, 16, 19] to > 6000 [13] participants with CRC. The lack of replication may also be driven by differences in specific outcomes investigated and clinical characteristics such as stage, tumor location, treatment modalities (e.g., chemotherapy, surgery, radiation), and microsatellite instability. Because these factors may influence mortality outcomes and the biological pathways that drive tumor progression and mortality, they may influence the SNP estimates obtained from GWAS leading to heterogeneity between studies [20].

Differences in genetic ancestry between studies may also impact replication, as individuals with African ancestry have different patterns of linkage disequilibrium and minor‐allele frequency compared to individuals with predominantly European or Asian ancestry [22]. Black‐identifying individuals in the United States are also more likely to be diagnosed at an advanced stage and have more aggressive tumor characteristics [72], as well as higher levels of specific mortality risk factors such as obesity and diabetes [73, 74], which may influence the impact of genetic background on CRC survival [20]. Consequently, genetic associations identified in these European‐ or Asian‐ancestry samples may not replicate in African‐ancestry individuals. For these reasons, it is critical to identify genetic variants associated with CRC mortality among African‐ancestry individuals, so that all populations can benefit from advances in precision medicine. Importantly, results from the present study may not generalize across diverse populations and require replication in an independent sample. In a study of 358 African American individuals receiving treatment for Stage II–III colon cancer, Penney and colleagues report an association between chromosome 7 variant rs76766811 and risk for overall mortality (per allele HR 2.82, CI: 1.88–4.23) [14]. The authors note that this variant lies downstream of *SKAP2*, a gene implicated in immune system activation that is linked to tumor macrophage infiltration and cancer progression [75]. We do not observe an association for this SNP with mortality outcomes in the present sample, and none of the statistically significant associations we identify were from Chromosome 7. Lack of replication in the present sample may be due to differences in study design, as not all participants were actively receiving chemotherapy at the time of diagnosis.

Strengths of our study include enrichment for individuals with African ancestry, who have unique genetic characteristics but are underrepresented in genetic research. Further, the SCCS sample includes a majority of participants with low socioeconomic status, who are also underrepresented in epidemiologic research despite having elevated mortality risk. Lastly, we utilize genomic imputation to address missing genotypes, which drastically improves genomic coverage and makes it possible to identify associations for SNPs that were not directly genotyped [33]. Although there are challenges imputing rare variants, previous research demonstrates that imputation accuracy is satisfactory for variants with MAF > 0.05 [33], which was the threshold used in this study. SNPs with poor imputation quality (*R* ^2^ < 0.30) were excluded from the analysis. Limitations of our study include the small overall sample size, which likely limits the statistical power to identify SNPs with small or modest impacts on mortality outcomes. Sample sizes are especially limited for stratified analyses, and consequently, the observed effect modification by tumor location warrants corroboration in an independent sample. Likewise, although we attempt to replicate our findings in an independent sample of Black‐ or African American‐identifying individuals with CRC from the TCGA, the replication sample size is limited (*N* = 65), and consequently, independent replication in a larger cohort is still required. Information about cancer treatment was limited and consequently not included in the analysis. While we exclude participants with an anal cancer diagnosis, it is possible that some participants identified via the NDI died from anal cancer rather than CRC, contributing to heterogeneity. Also, we do not report associations for rare variants, which may have a significant impact on cancer outcomes but remain difficult to identify in GWAS due to statistical power. Likewise, we do not perform analyses stratified by tumor stage or treatment status, which limits comparability to previous studies. While there is the potential for batch effects given the number of genotyping platforms used, we attempt to control for this by adjusting for the principal components. There may also be residual population stratification despite adjustment for principal components. It is possible that the GWAS estimates are impacted by survival bias as we are unable to account for the impact of the variants on CRC risk, although we expect this bias to be minimal as none of the reported variants have been linked to CRC incidence or any other disease outcomes. Lastly, the GWAS study design does not allow for the identification of causal variants, which may be unmeasured.

## 5 Conclusion

We report novel associations between germline genetic variants mapping to *LTBP1* and *MCTP2* and CRC mortality outcomes in a sample of individuals with Black racial identity who developed CRC. These findings may support the development of precision medicine approaches to manage CRC across diverse populations, or novel interventions targeting biological pathways that influence cancer development and progression. However, additional research is required to corroborate these results in an independent cohort.

## Author Contributions

**Thomas Lawler:** formal analysis, investigation, methodology, writing – original draft. **Jirong Long:** data curation, writing – review and editing. **Rene Welch:** formal analysis, methodology, writing – review and editing. **Irene Ong:** writing – review and editing. **Oluwatoyosi Ogunmuyiwa:** writing – review and editing. **Rida A. Khatri:** writing – review and editing. **Martha Shrubsole:** resources, writing – review and editing. **Shaneda Warren Andersen:** conceptualization, funding acquisition, methodology, project administration, resources, supervision, writing – review and editing.

## Ethics Statement

All study activities conformed to the Declaration of Helsinki. The Institutional Review Boards of Vanderbilt University Medical Center and Meharry Medical College provided approval for this study.

## Consent

All participants provided written informed consent.

## Conflicts of Interest

The authors declare no conflicts of interest.

## Supporting information


**Figure S1:** QQplots for genome‐wide association analysis of overall and cancer‐specific mortality among individuals with incident CRC (*N* = 500).

**Table S1:** Summary of associations between single‐nucleotide polymorphisms and CRC‐specific mortality (pvalue < 5.0 × 10^−6^). **Table S2:** Summary of associations between single‐nucleotide polymorphisms and overall mortality (*p*‐value < 5.0 × 10^−6^).

**Table S3:** Summary of associations between single‐nucleotide polymorphisms and overall mortality among individuals with colon or rectal adenocarcinoma and self‐identified Black or African American race from The Cancer Genome Atlas (N = 65).

**Table S4:** Summary of previously reported genome‐wide associations for overall or colorectal cancer‐specific survival outcomes obtained from the NHGRI‐EBI GWAS catalogue.

**Table S5:** Summary of previously reported genome‐wide associations for colorectal cancer progression or recurrence outcomes obtained from the NHGRI‐EBI GWAS catalogue.

## Data Availability

The data that support the findings of this study are available from the Southern Community Cohort Study, but restrictions apply to the availability of these data, which were used under license for the current study and are not publicly available. Data are, however, available from the authors upon reasonable request and with permission of The Southern Community Cohort Study. Study code is available from the corresponding author upon reasonable request.
