# Supplementary figures and images for: A Genome‐Wide Association Study of Colorectal Cancer Mortality Outcomes Among Individuals of African and Admixture Ancestry
Source: Mol Carcinog. 2026 Jan 29;65(4):422–33. doi: 10.1002/mc.70086 (PMC12973162; doi:10.1002/mc.70086)

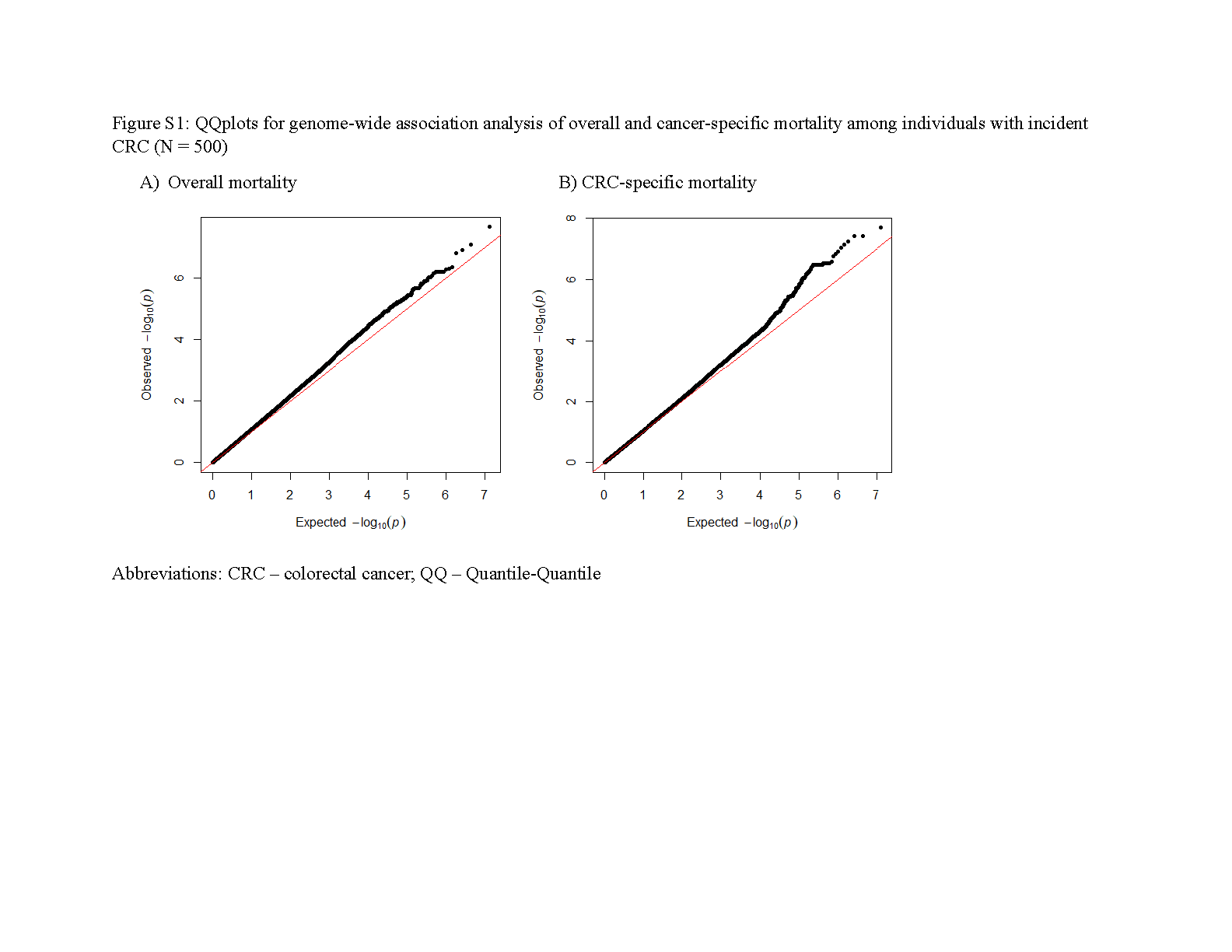

Supplement: Supplementary file 1 — Figure S1: QQplots for genome‐wide association analysis of overall and cancer‐specific mortality among individuals with incident CRC (N = 500). [file MC-65-422-s004.tif]
