# Supplementary material for: A Genome‐Wide Association Study of Colorectal Cancer Mortality Outcomes Among Individuals of African and Admixture Ancestry
Source: Mol Carcinog. 2026 Jan 29;65(4):422–33. doi: 10.1002/mc.70086 (PMC12973162; doi:10.1002/mc.70086)
Supplement: Supplementary file 3 — Table S3: Summary of associations between single‐nucleotide polymorphisms and overall mortality among individuals with colon or rectal adenocarcinoma and self‐identified Black or African American race from The Cancer Genome Atlas (N = 65). [file MC-65-422-s001.docx]

| **Table S3:** Summary of associations between single-nucleotide polymorphisms and overall mortality among individuals with colon or rectal adenocarcinoma and self-identified Black or African American race from The Cancer Genome Atlas (N=65) | | | | | | |
| --- | --- | --- | --- | --- | --- | --- |
| **rsID^*^** | **Chr:position^*^** | **Gene^*^** | **Alleles**  **(major/minor)** | **Sample MAF** | **HR^†^**  **(95% CI)** | **P-value** |
| rs34071846 | 2:33314964 | *LTBP1* | C/A | 0.05 | -^‡^ | - |
| rs12712337 | 2:33315230 | *LTBP1* | C/T | 0.05 | -^‡^ | - |
| rs10103953 | 8:8834240 | *LOC124901866* | G/T | 0.45 | 4.45 (1.22-16.26) | .02 |
| rs7171579 | 15:94838899 | *MCTP2* | C/T | 0.35 | 0.65 (0.25-1.66) | .37 |
| ^*^rsIDs and variant chromosome, position, and gene are from the Genome Reference Consortium human reference genome GRCh37.  ^†^All hazard ratios are per one minor allele increase, adjusted for age at diagnosis, sex, and stage  ^‡^Hazard ratios not estimable  Abbreviations: CI – confidence interval; CRC – colorectal cancer; HR – hazard ratio; MAF – minor allele frequency | | | | | | |
